# The Lineage Theory of the Regional Variation of Individualism/Collectivism in China

**DOI:** 10.3389/fpsyg.2020.596762

**Published:** 2021-01-20

**Authors:** Weigang Gong, Meng Zhu, Burak Gürel, Tian Xie

**Affiliations:** ^1^Department of Sociology, Wuhan University, Wuhan, China; ^2^Institute for Advanced Studies in Finance and Economics, Hubei University of Economics, Wuhan, China; ^3^Department of Sociology, Koç University, Istanbul, Turkey; ^4^Department of Psychology, Wuhan University, Wuhan, China

**Keywords:** individualism, collectivism, cross-cultural psychology, rice theory, climato-economic theory, Chinese culture, Chinese lineage

## Abstract

China has undergone a rapid process of modernization since 1949. The modernization process has accelerated with the development of the market economy and rural-to-urban migration after the 1980s. Nevertheless, Chinese regions still exhibit substantial differences in terms of individualist/collectivist cultural orientations. The rice theory and the climato-economic theory have attempted to explain this variation by analyzing provincial-level data. Based on a quantitative analysis of more granular, county-level variables spanning from the early 1990s until 2010, we offer an alternative account of this cultural variety based on lineage development in different Chinese regions. Using the ArcGIS geographic information system, we first present the regional distribution of individualism/collectivism indicators at the county level through descriptive statistics and spatial analysis. We also run a regression model to analyze county-level data on individualism/collectivism that includes three periods (1990, 2000, and 2010). Our multi-level analysis shows that lineage development is a critical variable that explains more regional variation of culture in China when compared to other variables. While rice farming, the key variable of the rice theory, is a significant variable, its explanatory power is less than the lineage variable. Finally, our analysis shows that the climato-economic theory fails to explain the regional variation of culture.

## Introduction

The existing literature identifies industrialization, economic growth, urbanization, and population mobility as leading socio-economic factors behind the rise of individualism in China (Yan, 2009). China has a history of 5,000 years, and agricultural production and village settlements have remained their defining characteristics for over 2,000 years (termed as the “agricultural era”). Although Chinese society entered the modernization era by the nineteenth century (at the latest), the pace of modernization significantly increased after the foundation of the People’s Republic of China in 1949, especially in rural areas that account for the vast majority of the population. Development of the market economy and rural-to-urban migration occurred after 1978 and sped up in the 1990s (a short period compared to the long history of China).

This paper explores whether the significant regional variation of ecology and social history in the pre-modern period has any effect on the regional variation of individualism/collectivism (I/C hereafter) in contemporary China. We argue that the impact of modern individualist culture has been uneven in different Chinese regions due to their long-held differences in the development of lineages. In other words, China’s regional variation of I/C has resulted from regional differences in the degree of the lineage development. To ground our argument empirically, we analyze a wide variety of longitudinal historical data from the post-1949 period.

## Literature Review

Individualism and collectivism are central categories of cross-cultural psychology (Triandis et al., 1988). When interacting with others within a community, individuals can take two opposite directions: maintaining personal autonomy or entirely integrating into the community. Each cultural entity is located somewhere between these two poles. Within individualist cultures, values and ethics support personal autonomy and interest. Individuals tend to be self-dependent and do not need emotional support from the community. Conversely, within collectivist cultures, the community takes priority over individuality, and collective interests prevail over individual interests. Personal identities are based on group membership, and groups are responsible for their members’ well-being.

Individualism and collectivism constitute two endpoints of the same theoretical dimension (Triandis et al., 1988; Oyserman et al., 2002; Hofstede, 2011). Different types of cultures are located at different places along this continuum. I/C can be assessed within group dynamics. Following the beginning of humankind, family and community emerged as the first and second collectivities, respectively. Extended family was dominant in some cultures, while the nuclear family was dominant in others. Collectivism develops relatively easily in the former, and individualism develops more easily in the latter. Individuals acquire one of these two tendencies during adolescence, after which it is strengthened through schooling and other organizational experiences. As a result, intercultural differences are gradually solidified.

The conceptualization of I/C as a continuum between extreme individualism and extreme collectivism has helped the scholars of cross-cultural psychology to compare not only individualist and collectivist cultures but also the I/C tendencies within each (collectivist or individualist) group. Psychologists tend to view I/C as two dimensions of analysis. Starting from the individual level, they analyze the relationship between the individual and the community. Based on the individual-community relationships within different cultures, they assess the collectivist and individualist tendencies within each cultural entity (Triandis et al., 1988; Oyserman et al., 2002).

Hofstede (2011) explains the spatial variation of I/C based on differences in geographic latitude, climate, and wealth. Berry (1976) explains it based on historical, geographical, and ecological variables, developing a theory based on differences in survival methods. Survival theory suggests that some survival methods (such as farming) require more cooperation (e.g., the construction and maintenance of water conservancy systems). Such practices lead to the formation of a more collectivist cultural orientation in the long run. In contrast, since individualized forms of action are more suitable for other ways of survival, such as nomadism and hunting, they contribute to the formation of more individualist cultural orientations (Berry, 1976).

On the other hand, the causal analysis of cultural differences is a challenging endeavor. One of the problems in existing survival theories is that when explaining cultural orientation from the perspective of survival methods, it is difficult to control the role of religion, politics, history, and many other factors. Analysis of the differences in I/C among different regions within a single cultural and political body has recently emerged as a research frontier in cross-cultural comparative psychology. For example, Varnum and Kitayama (2011) compared the individualist tendencies of different regions in the United States, while Yamawaki (2012) conducted a similar comparative analysis in Japanese regions.

As a large country with a history of 5,000 years and 9.6 million km^2^, China exhibits vast regional differences in ecology, history, and cultural psychology. A growing body of scholarship informed by I/C theories investigates cultural differences among different regions of China (Van de Vliert et al., 2013; Talhelm et al., 2014). However, existing accounts of the regional variation of collectivism and individualism in China seriously contradict each other. Hence, further empirical analysis and theoretical conceptualization are needed.

### The Climato-Economic Theory of I/C in China

Van de Vliert et al. (2013) explain the regional variation of I/C in China from the perspective of the climato-economic theory of culture. According to this theory, 22°C is the most suitable temperature for human survival; the more it deviates from this number, the greater are the challenges of survival. Resource constraints caused by unsuitable temperatures (too high or too low) compel humans to cooperate in the production of food and other essential items. In contrast, the ease of obtaining food and other necessities keeps the cooperation imperative at a minimum. The situation also changes in areas with inhospitable climates. As long-term cooperation decreases challenges of survival and increases wealth over time, the cooperation imperative correspondingly declines. Based on this theoretical framework and a provincial-level analysis of the survey data, Van de Vliert et al. (2013) claim that collectivism is the weakest in southern regions with temperate climates (irrespective of income), such as Guangdong; is a bit stronger in higher-income provinces with challenging climates, such as Hunan; and is the most robust in poor provinces with challenging climates, such as Inner Mongolia, Xinjiang, Tibet, Qinghai, and Heilongjiang.

### The Rice Theory of I/C in China

Talhelm et al. (2014) explain the cultural diversity of Chinese regions based on the survival theory. According to this theory, a more collectivist culture has emerged over the long history of rice cultivation in South China because rice requires more irrigation, thereby necessitating greater cooperation among cultivators. In contrast, a significantly lower degree of labor cooperation has been the norm in North China (where wheat has been the dominant crop) because wheat growing does not require much irrigation, leading to a relatively independent and more individualist culture. Based on these observations, Talhelm et al. (2014) claim that collectivist culture has been more influential in southern regions such as Fujian, Guangdong, Guangxi, Shanghai, and Zhejiang. In contrast, individualism has been dominant in northern provinces (such as Henan, Shandong, Shanxi, and Shaanxi) and northeastern provinces (such as Heilongjiang and Liaoning).

The two theories discussed above make squarely opposite arguments with regard to several regions. For example, the climato-economic theory asserts that in Guangdong, Fujian, Jiangxi, Hainan, and other southeastern regions, the collectivist cultural tendency is the weakest. However, the rice theory predicts the opposite, arguing that these same areas (where rice farming is most prevalent) contain the strongest tendencies for collectivism in China. Moreover, the climato-economic theory argues that the three northeastern provinces (Heilongjiang, Jilin, and Liaoning) contain collectivist tendencies, while the rice theory views them as regions with individualist tendencies (Table 1). Apparently, further study is imperative in analyzing regional variations of collectivism and individualism in China, as well as the critical factors affecting this difference. The contradictory conclusions of the climato-economic theory and rice theory are not simply due to problems of measurement but rather likely result from their neglect of important factors that shape regional variations of collectivism and individualism in China.

**Table 1:** Regional variation of individualism/collectivism (I/C) in China.

	Individualist tendency	Collectivist tendency
Talhelm et al., 2014	Anhui, Beijing, Gansu, Guizhou, Hebei, Henan, Heilongjiang, Jilin, Liaoning, Ningxia, Shandong, Shanxi, Shaanxi, Tianjin, Yunnan, and Qinghai	Fujian, Guangdong, Guangxi, Hainan, Hubei, Hunan, Jiangsu, Jiangxi, Shanghai, Sichuan, Chongqing, and Zhejiang
Van de Vliert et al., 2013	Hunan, Jiangsu, Beijing, Chongqing, Guizhou, Sichuan, Fujian, Zhejiang, Tianjin, Yunnan, Guangdong, Shanghai, Guangxi, Ningxia, and Hainan	Heilongjiang, Jilin, Liaoning, Inner Mongolia, Qinghai, Shanxi, Tibet Autonomous Region, Gansu, Hebei, Shaanxi, Xinjiang, Shandong, Hubei, Henan, Jiangxi, and Anhui

*Talhelm et al.’s study does not investigate Xinjiang, Tibet, and Inner Mongolia*.

We provide an alternative explanation of the same phenomenon based on the historical differences in the power of lineage organizations among Chinese regions. Below, we present our findings regarding China’s regional differences in individualism and collectivism and discuss the lineage-based theory that accounts for these differences.

The problems of the climato-economic theory and rice theory are partly related to their units of analysis. They use provinces as units of analysis to explain the regional variation of cultures in China. However, this unit of analysis does not explain such variation in Chinese culture because many of the provinces include several sub-regions with significantly different cultural norms and values. Historically, the cultural heterogeneity of Chinese provinces is related to the principle of the jigsaw-like drawing of provincial boundaries occurring since the Yuan Dynasty period (1271–1368). Including different natural regions in a single province was an intentional policy designed to check centrifugal forces, prevent large-scale rebellions, and enhance the power of the central state. Since provincial boundaries, by design, have functioned to hide real similarities and differences among regions, the province cannot be used as a unit to analyze regional variations in Chinese culture.

### Lineage-Based Framework of the Regional Variation of I/C in China

In his comparative analysis of lineage organizations in the Fujian and Guangdong provinces, Maurice Freedman (1958) made one of the earliest attempts at delving into the regional variation of lineage development in China. Freedman defined the Chinese lineage as a kinship-based economic and political organization, stressing that lineage power was regionally varied. A single lineage typically encompasses the entire village population in the Fujian and Guangdong provinces; in other words, lineage and village tend to coincide. Lineage organization is less potent in the rest of China, as lineage typically comprises only one section of a village’s population. In his comparative analysis of Chinese lineages, Indian castes, and American clubs, renowned Chinese anthropologist Hsu (1963) argued that the effect of these three institutions on people’s lives is similar. According to Hsu, family and lineage – essentially indicating a significantly expanded variety of family – are the leading social organizations through which people solve their problems. Both manifest the mutual dependence in interpersonal relations and reproduce the social basis of a collectivist culture. They also cultivate a worldview centered on group spirit. The Chinese people growing up in this kind of environment cherish the companionship, security, and status associated with family and lineage. Even after years of work outside their hometowns, Chinese people are inclined to return and spend the rest of their lives with their family and lineage members. According to Hsu (1963), lineage culture and collectivist behavior are closely related. Notwithstanding the merit of his work, Hsu’s most critical shortcoming is the absence of inquiry into the regional variation of lineage development in China.

We argue that the lineage theory of culture explains the regional variation of Chinese culture more accurately than the climato-economic and survival theories. Some academic studies emphasize the importance of lineage development in the cultural diversity of Chinese regions, but, so far, this strand of scholarship has not presented a case based on quantitative data analysis. In a close reading of the historical literature on the subject, Wang, 2006 and He (2012) explain the regional variation of lineage development, taking into account historical factors such as war, migration, and ecological/environmental factors. According to Wang and He, earlier population settlement and relatively stable socio-political circumstances have allowed the development of a strong collectivist culture built around large lineages in rural South China. While population settlement occurred relatively late in North China, where its occupants suffered from frequent wars for centuries, the stabilization of socio-political conditions since the foundation of the Ming dynasty allowed the North to inherit the family culture of the Han people in the Central Plains and develop powerful lineage structures, albeit not as strong as those in the South. Finally, compared to these two macro-regions, the Yangzi River Basin (including southern Anhui, the Dongting Lake Plain, the Jianghan Plain, and the Chengdu Plain) was settled by migrants from various parts of China escaping from economic hardship and political instability. Since the duration of a stable human settlement was relatively short and lineage structures were, for the most part, underdeveloped, a relatively individualist culture prevailed even before the twentieth century but has become even stronger since the 1980s (Freedman, 2000; Wang, 2006, 2007; He, 2012).

The Chinese are generally believed to have a cultural tradition of collectivism. In the traditional Han culture, the interests and goals of the individual are subordinate to the goals of the family and lineage. The Chinese pay respect to their ancestors by burning incense, which is believed to help the family flourish and protect itself. The practice of “co-residence and common property” – in which different generations live in the same household and share their income – is the primary manifestation of a collectivist culture. Such a collectivist cultural orientation permeates all aspects of traditional Chinese life (Shiga, 2003), and the culture expresses itself through patterns of reproduction, marriage, and childbirth.

In the family reproduction model, the married child’s family is still part of the mother’s family, further expanding the definition of family (Fei, 2001). According to the collectivist principle of “co-residence and common property,” adult sons usually live with son’s father’s home, and the income of all family members is considered as a collective income. If a newlywed child attempts to become independent through separation from the family, it is seen as a sign of family disunity and is often opposed by parents and other brothers (Wang, 2006). In the fertility model, the inevitable result of pursuing more children and passing on lineages is high fertility and patriarchy. In the marriage model, marriage is not meant for individual happiness but for the continuation of the family and raising children. Unmediated, autonomous marriage based on romantic love between two individuals is not supported, and if a woman is not happy in her marriage, she cannot easily file for divorce.

With the rise of individualism brought about by China’s modernization, citizens place more importance on self-realization and the pursuit of individual happiness, and traditional ideas about reproduction and other collectivist goals are increasingly being abandoned. Hence, although there are still many obligations among family members, married children will become more independent from their parents’ families through separation so that they can independently control the property of their smaller family without having to live with parents and other siblings (Yan, 2002).

Due to the rise of individualism in China, the continuation of the patrilineal family and lineage prosperity is no longer relevant, resulting in a sharp decline in fertility. In the marriage model, marriage is increasingly based on romantic love between young men and women. The pursuit of individual happiness leads young couples to reject unhappy marriages, resulting in a sharp increase in China’s divorce rate since the 1990s. The individualist tendency, however, is not only manifested in the choice of divorce and having fewer children. It is also reflected in the arrangement of individual property rights, family residence (to protect personal privacy), and the individualization of intergenerational relationships (Yan, 2012).

## Materials And Methods

### Hypothesis

To assess the validity of our argument that the regional variation of lineage development is a key factor behind the regional variation of I/C in China, we tested the following research hypothesis:

**H1:** The regional variation of lineage culture corresponds with the regional variation of I/C.
**H1a:** The stronger the lineage development, the stronger the collectivist cultural tendency.
**H1b:** The weaker the lineage culture, the stronger the individualistic cultural tendency.

### Measurement of I/C at the County Level

The works of Triandis et al. (1988) and Hofstede (2011) show that family is a fundamental unit used to operationalize and measure the concepts of I/C. In *Culture’s Consequences*, Hofstede frequently stresses that the family is the first inner group that people come into contact with and growing up in different types of families leads to significant differences in the psychological and behavioral tendencies of individuals. Those who are raised in extended families are more inclined to regard themselves as part of the family and adjust their individual goals to conform to the family’s goals, thereby maintaining a distinctly collectivist tendency. In contrast, those who are raised in nuclear families tend to think of the family as a combination of different individuals so that individual goals take precedence over family goals, thereby maintaining a more individualistic tendency. Among Hofstede’s various scales measuring I/C, the family scale appears the most significant. Hence, we focus on the family as the primary unit used to measure I/C.

Other scholars have also adopted the measurement of I/C from the perspective of family. For example, Vandello and Cohen’s index of collectivism in the United States (1999) includes the following eight indicators: the percentage of people living alone, the ratio of people carpooling to work to driving alone, the ratio of divorce rate to marriage, the percentage of elderly people living alone, the percentage of households containing grandchildren, the percentage of people with no religious affiliation, the average percentage of Libertarian votes over the last four presidential elections, and the percentage of self-employed people (Vandello and Cohen, 1999). They found that collectivist tendencies were strongest in the Deep South, while individualist tendencies were strongest in the Mountain West and Great Plains.

Yamawaki (2012) used Vandello and Cohen’s index as a model to develop a collectivism scale suitable for a prefecture-level analysis in Japan. This scale includes five indicators: the divorce-to-marriage ratio, the percentage of elderly living alone, the percentage of nuclear families, the percentage of individuals living alone, and the percentage of three-generation households. Yamawaki found considerable variations of collectivism within Japanese culture. The majority of collectivistic prefectures is in northern Japan, whereas the most individualistic prefectures, although spread across the country, are typically located in the most urbanized areas.

The present study uses Yamawaki’s five-index method to measure I/C. To measure each Chinese county’s level of I/C, we use the data from China’s Fifth (2000) and Sixth (2010) Population Censuses. This data include numbers regarding divorce rates, proportions of individuals living alone, percentages of three-generational families, and family size. To more precisely measure the individualistic/collectivistic tendency within a family, we formulated an “independence of married youth” indicator that measures the proportion of married young people 25–35 years of age who are heads of their families in the corresponding population cohort. In other words, the stronger the independence of young offspring, the more likely they are to become independent from their parents after marriage and establish their own nuclear family.

Our analysis includes two additional variables – fertility rate and preference for sons. The existing literature (Gong et al., 2013) indicates that collectivist Chinese families have more children and prefer sons to maintain the family. However, with the rise of individualism in China since the 1980s, more people seek individual enjoyment and self-actualization as their main life goal rather than the continuation of the family. Greater individualism, therefore, leads to a decline in fertility and preference for sons.

### Measurement of Lineage Development at the County Level

The existing literature measures the degree of lineage development based on surname structure and surname concentration (Tsai, 2002, 2007; Peng, 2009, 2010; Guo and Yao, 2014). The 2005 national survey conducted on 1% of the population sample provides the county-level surname data of the participants, which enabled us to assess the degree of lineage development in different Chinese regions by calculating the concentration of surnames.

### Measurement of the Cropping Pattern at the County Level

In their article advancing rice theory, Talhelm et al. (2014) measure the cropping pattern in China based on each county’s rice planting ratio. We measure the cropping pattern in the same way. In a recent article, Talhelm et al. (2018) claims that historical data on rice farming can better isolate the impact of urbanization on I/C patterns and, therefore, has more power to predict I/C. Data on the proportion of rice cultivation in more than 3,000 counties of China are obtainable only from large-scale statistical databases such as agricultural censuses. Therefore, we use the data of China’s first agricultural census carried out in 1996 to measure the proportion of rice cultivation at the county level. This census contains the earliest available data on the cropping pattern at the county level. China’s rapid urbanization process began by the early 1990s, but according to the World Bank (n.d.), only a quarter of the total population was urban in 1990. Hence, the impact of urbanization on planting patterns was relatively limited at the time of this census. Moreover, China joined the World Trade Organization in 2001, so the effect of international trade on planting patterns was also minimal. Therefore, we believe that the 1996 agricultural census data are a better resource for measuring China’s planting patterns.

### Measuring the Climatic Demands-Income Resources at the County Level

In their article advancing the climato-economic theory, Van de Vliert et al. (2013) use temperature and GDP per capita data. We obtained temperature information at the county level and used the per capita GDP of each Chinese county. Since Van de Vliert et al. stress the interactive effects of climate and wealth, we used the interaction terms between the per capita GDP and temperature of each county in China for 2000 and 2010.

#### Climatic Demands

In line with past research (e.g., Van de Vliert et al., 2013), climates are considered as more demanding when temperatures in the coldest and hottest months deviate from 22°C. Specifically, climatic demands (CDs) were operationalized as the sum of the four absolute deviations from 22°C for the average lowest and highest temperatures in January and July (data available for the capital city of each county from http://www.resdc.cn/data.aspx?DATAID=288).

#### Income Resources

For each county, the per capita GDP of 2000 and 2010 was taken from www.stats.gov.cn. Income resources (IRs) and CDs were insignificantly related predictors.

### Data

We collected county-level data on the degree of lineage development, the ratio of rice planting, climate demand-income resources (CD-IRs), the rate of urban population, and I/C variables. We used the concentration of surnames as the indicator of lineage development at the village and county levels. We extracted the county-level lineage development data from China’s 1% population sample survey (conducted in 2005) that provides data on the surnames of Chinese people. To measure the level of lineage development, we used this database to calculate the concentration of surnames in more than 3,000 Chinese counties. Using the Chinese General Social Survey (2006) data, we measured the level of lineage development by calculating the proportion of the top three surnames in the total population of administrative villages.

#### Rice Planting Ratio

Rice planting ratio, the ratio of rice fields within all cultivated land in a given region, is the primary explanatory variable of rice theory and is obtainable for over 3,000 Chinese counties from the national agricultural census data. The earliest national agricultural census was conducted in 1996 and provides data on the proportion of rice, wheat, corn, and other crops in the total cultivated area. We believe that the ratio of arable land used for growing rice is close to the proportion of paddy fields in each county because rice is a staple crop in China. We mainly used the statistical yearbooks of various provinces and statistical yearbooks of prefecture-level cities to collect data on the proportion of rice cultivation in each county at the time of the first agricultural census in 1996 (Tongji Nianjian Fenxiang Pingtai, n.d.a).

#### Climate Demand-Income Resource

To calculate CD at the county level, we needed to obtain temperature information in January and July for more than 3,000 counties in China. We, therefore, purchased 12-month temperature information from more than 3,000 counties for the year 2000 from the China Meteorological Administration. To calculate the CD factor, we first calculate the absolute value of the deviation of each county from 22°C in January and July and then sum up these two values. The IR factor data comes from the data on GDP per capita in the regional statistical yearbooks of various regions in China for 2000 and 2010. The statistical yearbooks of various provinces and prefecture-level cities are accessible online (Tongji Nianjian Fenxiang Pingtai, n.d.a).

#### Urban Population

We calculated the data on the proportion of the non-agricultural population based on the original data from the 2000 and 2010 censuses (National Bureau of Statistics of China, 2000, 2010).

The fifth and sixth census data also include many variables that measure individualism and collectivism at the county level. The variables such as divorce rate and the percentage of single-person households measure individualism, while the variables such as the preference for sons, the percentage of three-generation families, fertility rate, and family-size measure collectivism.

We utilize 1990, 2000, and 2010 population census data (Tongji Nianjian Fenxiang Pingtai, n.d.b; National Bureau of Statistics of China, 2000, 2010) to measure divorce rate, fertility rate, sex ratio at birth, proportion of three-generation households, and young children’s level of independence at the county level. Similarly, we also measure the degree of lineage development at the county level – the primary explanatory variable of this study – and control variables such as per capita GDP, rice farming, CD, income sources, and the rate of urbanization. We used the method of Van de Vliert et al. (2013) to measure CD factors. Since there are more than 3,000 counties in China, we also go beyond county-level analysis. For the convenience of discussion, we have divided China into four macro-regions: South China, North China, the Yangtze River Basin, and Northeast China. We discuss the characteristics and influencing factors of collectivism–individualism in each region.

We collected county-level data from 31 provinces of China. We dropped some counties due to missing data. Overall, we analyzed data from 1,907 counties. We also collected district-level data for Beijing, Shanghai, Tianjin, and Chongqing. There are more first-tier cities in China, such as Wuhan and Guangzhou. We plan to collect and analyze more data on such cities in our future research.

## Results

Our analysis contains two parts. In the first, based on I/C variables, we discuss the regional variations of I/C and explain its characteristics of spatial distribution.

### Regional Differences of I/C Reflected in the Divorce Rate

**Figure 1:**
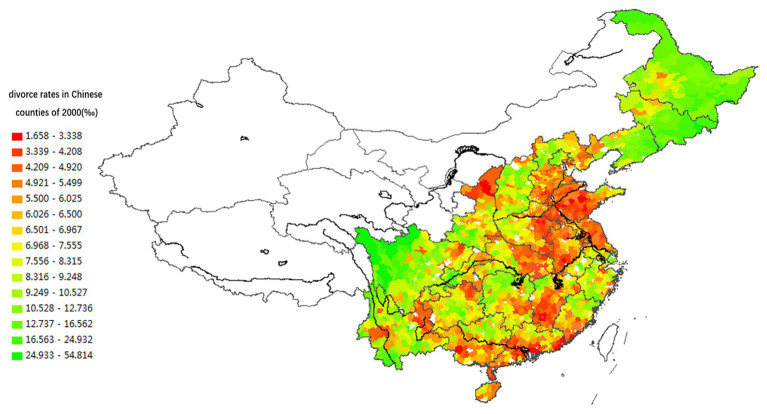
Spatial distribution of divorce rates in Chinese counties located to the east of the Aihui-Tengchong Line in the year 2000 (per thousand).

We use the ArcGIS geographic information system to explore the regional variation of divorce rate. We calculated “the logarithm of the number of divorced couples per 1,000 married couples” in the regions located to the east of the Aihui-Tengchong Line, where the Han Chinese constitutes the vast majority of the population (see Figure 1).^1^ The regional variation of divorce rates appears consistent in 2000 and 2010. Counties having similar divorce rates are adjacent rather than irregularly scattered. These neighboring counties form regions of considerable size, and the sex ratios at birth are also similar within each region.

**Figure 2:**
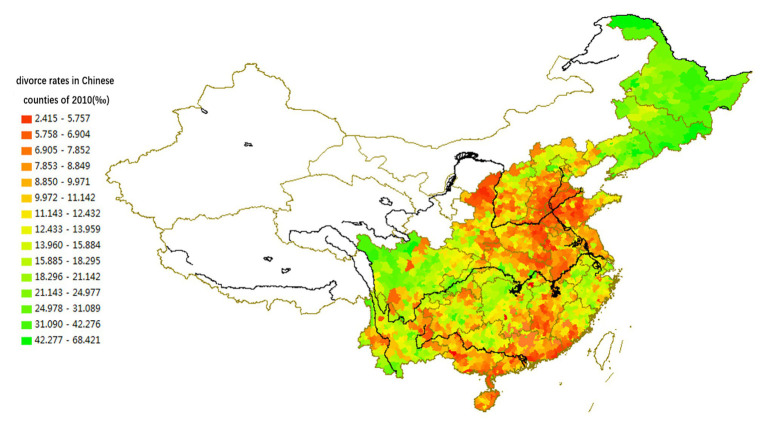
Spatial distribution of divorce rates in Chinese counties located to the east of the Aihui-Tengchong Line in the year 2010 (per thousand).

Figures 1, 2 demonstrate that the boundaries between regions containing different divorce rates often do not fit the provincial boundaries. A single province usually includes sub-regions with different characteristics; for instance, the northern and southern parts of Anhui, Fujian, Jiangsu, Hubei, and Hunan provinces have different divorce rates.

### Regional Differences of I/C Reflected in the Family Reproduction Model

The family reproduction model is a useful indicator for assessing the regional variation of I/C in China. A married son’s choice to either continue living with his extended family or set up his own nuclear family is critical for both his own life and the lives of his extended family members. The more independent the offspring, the more likely he is to begin his own household after marriage. If there is a stronger collectivist orientation in the family, the offspring is more likely to remain in the mother’s family long after marriage, abiding by the collectivist principle of “coresidence and common property.”

**Figure 3:**
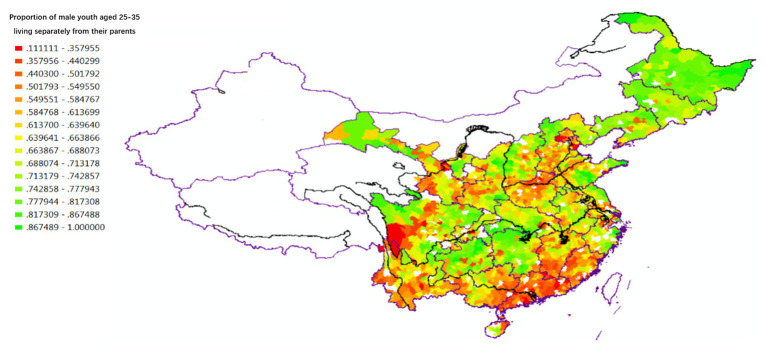
Independence of young children in intergenerational relations (1982).

**Figure 4:**
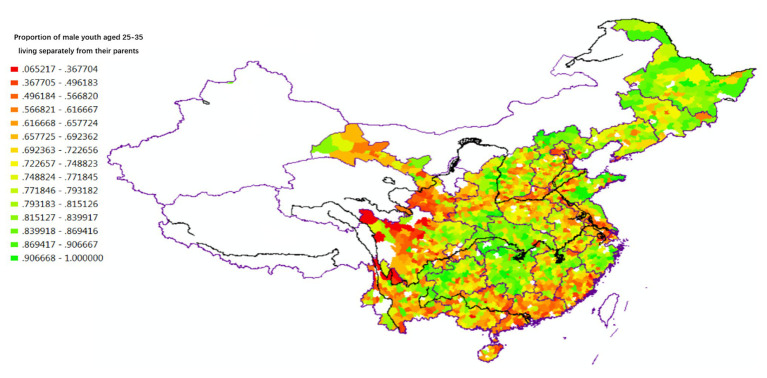
Independence of young children in intergenerational relations (1990).

Figure 3 shows that the children’s degree of independence after marriage varies between regions. In the early 1980s, before the rapid marketization and urbanization of Chinese society, married offspring living in the Yangzi River Basin and the Northeast (where individualist culture had been historically influential) were more inclined to separate from their parents’ families and establish their own independent nuclear families. In contrast, married offspring living in South China (where collectivist culture had been historically prevalent) were more inclined to continue living with their extended families, conforming to the principle of “co-residence and common property.” Analysis of the Fourth National Census (1990) data (see Figure 4) yields almost identical results with those of 1982, confirming the reliability of the household reproduction data for the analysis of regional variation in Chinese culture.

### Regional Differences of I/C Reflected in the Fertility Model

**Figure 5:**
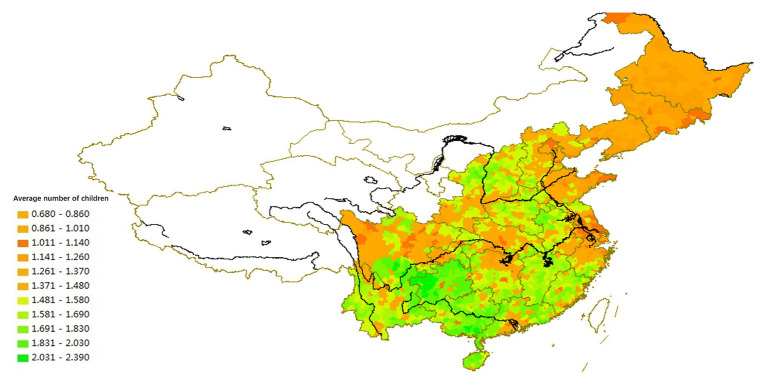
The spatial distribution of average number of births (2010).

**Figure 6:**
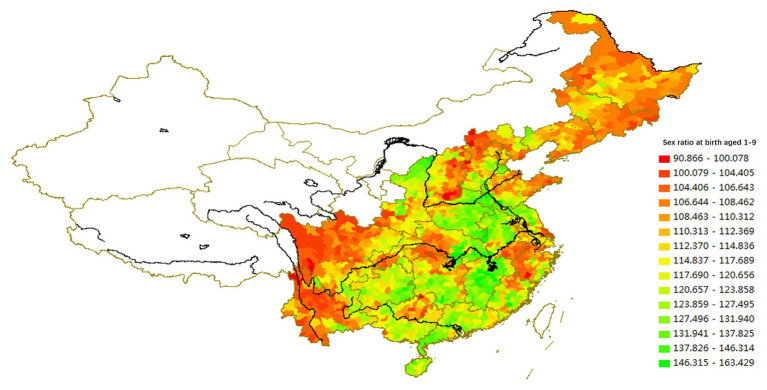
The spatial distribution of sex ratio at birth based on the Sixth National Census data (2010).

The fertility model includes two main dimensions: preference of the number of children and preference of gender, both of which are measured by the Chinese National Census. We analyze the national census data from 2000 to 2010 to illustrate the regional differences in fertility models (see Figures 5, 6).

In areas with a strong individualist culture, such as the Northeast, the Yangzi River Delta, the Jianghan Plain (southern Hubei), the Dongting Lake Plain (northern Hunan), and the Chengdu Plain, the fertility rate is the lowest in the country, and the preference for sons is weak. In areas with a strong collectivist culture, such as Guangdong, Fujian, southwestern Jiangnan, Henan, southern Hebei, southwestern Shandong, northern Anhui, and northern Jiangsu, the fertility rate is relatively high. These latter regions also have the highest sex ratio at birth in the country, reflecting the strong preference for sons.

### Regional Differences of I/C Reflected in the Intergenerational Relationship Model

Regional differences in I/C are also manifested in intergenerational family relationships. The traditional Chinese model of intergenerational relationships emphasizes the integration of father and son. The father provides all family resources to his children to help them to establish their own houses and businesses. In return, after their parents get old and retire, their children take care of them. Fathers and sons generally do not separate – that is to say, parents may separate from one or some of their sons, but they will not separate from all of their sons. It is rare for the elderly to live alone, especially in families with only one child; however, the rise of individualism has significantly changed this pattern. The concepts of individual property, personal privacy, and independent living have become popular. As a result, more parents live alone, and families are getting smaller.

**Figure 7:**
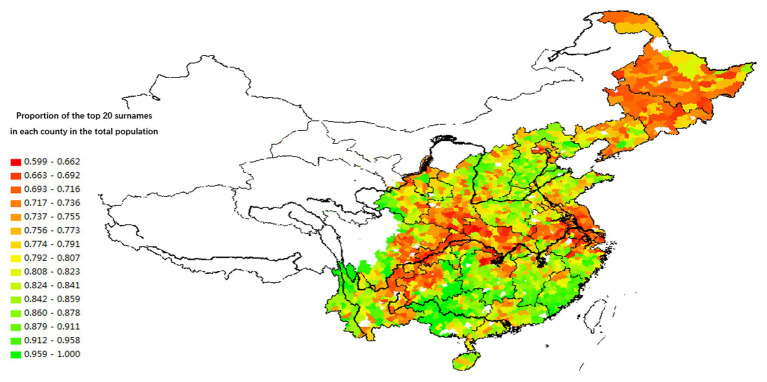
The spatial distribution of the proportion of the top 20 surnames in each county in the total population (2005).

**Figure 8:**
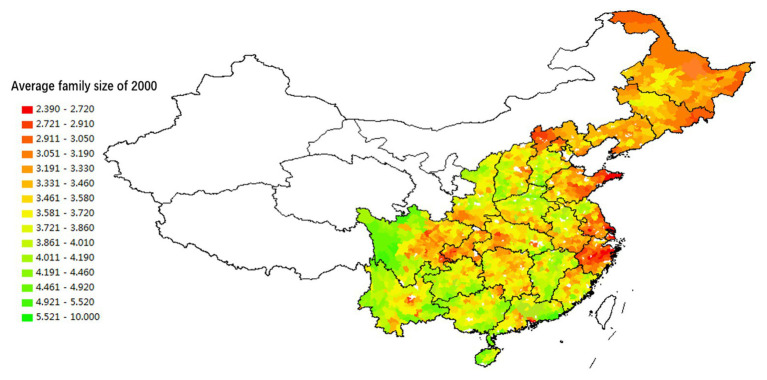
Spatial distribution of household size based on the Fifth Census data (2000).

Based on the data of the Sixth National Census conducted in 2010, we explore the regional variation of these transformations by using data on average family size to measure the degree of intergenerational cohesion and the nucleation of family structure. Figures 7, 8 show that the regional variation of family structure and intergenerational relationships are similar to those of divorce and fertility rates. Northeast China, the Yangzi River Basin, the Jianghan Plain, the Dongting Lake Plain, and the Chengdu Plain have relatively smaller family sizes. They also have the highest level of individualization of intergenerational relationships.^2^ The average family size is the largest in Guangdong, Fujian, Jiangxi, and other provinces in South China. This region also has relatively high intergenerational cohesion. Families in Henan, Northern Jiangsu, Northern Anhui, Central and Southern Hebei, Shanxi, and Shaanxi provinces in North China are similar to those in South China.

In short, we show that cultural differences among Chinese regions are reflected not only in divorce rates but also in fertility rates and model of intergenerational relationship. More specifically, we found that the spatial distributions of all three variables consistently overlap (see Table 2).

**Table 2:** Regional variation of I/C in China.

Region	Spatial scope	Child independence and intergenerational relationship			Fertility model		Marriage model (divorce rate)
		Family scale	Percentage of individuals living alone	Percentage of three-generation families	Sex ratio at birth	Fertility rate	Divorce rate
South China	Guangdong, Jiangxi, and Fujian	3.36	12.89	22.27	120.80	1.75	1.29
North China	Henan, Shandong, Shaanxi, southern Hebei, northern Anhui, and northern Jiangsu	3.22	11.04	19.44	118.22	1.49	1.17
Yangzi River Basin	Yangzi River Delta (Southern Jiangsu, southern Anhui and Shanghai); Hubei (Jianghan Plain); and Hunan (Dongting Lake Plain)	3.14	15.15	21.30	114.20	1.39	1.67
Northeast China	Heilongjiang, Jilin, and Liaoning	2.95	11.84	15.67	110.48	1.01	2.95
F Test		*F* = 17.8, *p* = 0.000	*F* = 69.9, *p* = 0.000	*F* = 97.4, *p* = 0.000	*F* = 33.8, *p* = 0.000	*F* = 178.1, *p* = 0.000	*F* = 57.8, *p* = 0.000

As shown below, the degree of lineage development that emerged in the long history of pre-modern China is a critical factor in explaining today’s regional differences in I/C. A strong individualist culture prevails in regions, where lineages are underdeveloped (such as the Northeast and most of the Yangzi River Basin), whereas a strong collectivist culture prevails in South China, where lineage culture has been influential.

The regional distribution pattern of I/C in China described above is different from that explained by the rice theory and the CD theory. One reason for this difference may be that using province as a unit of analysis (as these theories do) does not effectively illustrate regional variation in countries as large as China. Therefore, more micro-level units of analysis are needed. Based on large-scale data, our county-level analysis shows that counties in South China (Guangdong, Fujian, Jiangxi, Guangxi, southeastern Hubei, and southern Hunan) tend to have a collectivist cultural orientation (see Table 2). Second, the collectivist tendency, although not as strong in the South, is strong in North China (Henan, Hebei, Shandong, Shanxi, Shaanxi, and northern Anhui). In contrast, counties located in the Yangtze River Basin (the western part of the Sichuan Plain and the middle Yangtze river areas including Shanghai, southern Anhui, southern Jiangsu, the Dongting Lake Plain, and the Jianghan Plain – the core areas of Hunan and Hubei) have strong individualist tendencies. Similarly, individualism is strong in the Northeastern provinces (Heilongjiang, Liaoning, and Jilin).

### Measurement of Lineage Development in Different Areas

The most direct method to measure lineage development in different regions of China is to measure the proportion of the number of people carrying the same surname in the total population of a specific area. Decreasing the spatial scope increases the accuracy of this measurement. Increasing the spatial range increases the possibility of distortion because people with the same surname in a vast region may not have descended from a common ancestor. For example, the proportion of the population with several surnames in the total population of a village can accurately measure the development level of lineage because the population with the same surname in the village is most probably the descendants of the same ancestors. The earlier the first ancestors settled in the village, the higher the number of his children and grandchildren and consequently the higher the proportion of people with the same surname. Hence, the time period when the current pattern of population settlement was formed is closely related to the development of lineage organization and culture. Conversely, if the village’s settlement history is short, mature lineages are absent, the number of households with the same surname is small, and the distribution of surnames is relatively scattered.

The database of the Chinese General Social Survey (2006) contains variables that measure the degree of lineage settlement – that is, the proportion of the three largest surnames in the village to the total population and total number of village households. The data show that, at the administrative village level, lineage groups in South China (Guangdong, Jiangxi, Fujian, and Guangxi) are significantly larger than those in North China, which are in turn larger than lineages in Northeast and Southwest China. As Figure 8 illustrates, the concentration and power of lineages in South China are stronger than in North China, and those in North China are stronger than in Northeast China and the Yangzi River Basin. However, the micro-level data cannot directly represent the regional differences in lineage development.^3^

To assess the degree of lineage development across China, we expanded our unit of analysis, calculating the concentration of surnames in different regions based on the 1% sample survey in 2005. In China, the county has been a relatively stable administrative unit since the Qin and Han dynasties. In a typical core region of Han culture, there are more than a dozen surnames within the borders of a single county. The number of surnames found within its borders is often large if the county received a large number of migrants. Even if people with the same surname gathered in a county only by chance and without sharing similar descendants, the specific surname would not comprise a high proportion of the local population.

**Figure 9:**
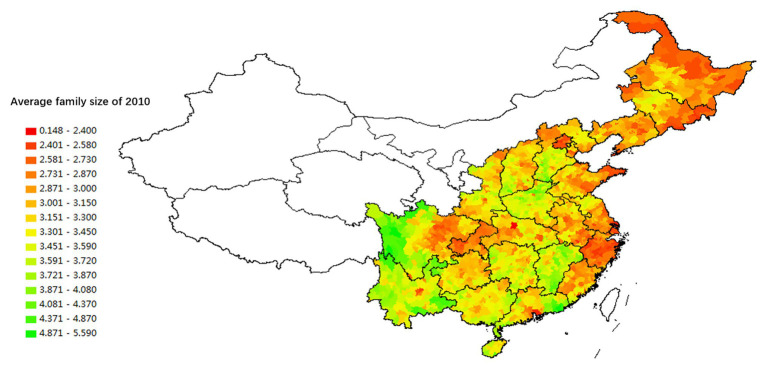
Spatial distribution of household size based on the Sixth Census data (2010).

The analysis results illustrated by Figures 8, 9 show consistency in the spatial distribution of surname concentration, both at the county and the administrative village level. When the surname concentration is high at the county level, it will also be high at the village level. That is, in South China counties with a high concentration of surnames, a typical village community often contains residents who share the same lineage. Most of the counties in Northeast China and the Yangzi River basin have relatively low surname concentrations, mainly because they received migrants from different regions and with different surnames.

Figure 9 also shows that the concentration of surnames in each county is not randomly distributed across China. Counties with similar surname concentrations are connected and form a relatively large area. For example, the three northeastern provinces and the Yangzi River Basin make up an area with a relatively low concentration of surnames, while areas with relatively high concentrations of surnames occupy large parts of North and South China. This finding confirms the long-term effects of regional variation in lineage development due to varied historical patterns of migration and settlement in China. The population settlement in Fujian, Guangdong, Guangxi, and Jiangxi provinces occurred earlier than in other areas, before the Song Dynasty. Those settlers often belonged to large families and nobles. A relatively peaceful and stable socio-political order in the following periods – marked by fewer wars and rebellions – was conducive to the development of lineage groups with identical surnames in South China compared to other Chinese regions. The higher concentration of surnames in North China (when compared to the Northeast and the Yangzi River Basin) can be explained by the North’s earlier population settlement. The Northeast and the Yangzi River Basin have the lowest concentration of surnames and the weakest lineage culture because of comparatively late population settlement, which did not allow the full development of lineage-based communities.

**Figure 10:**
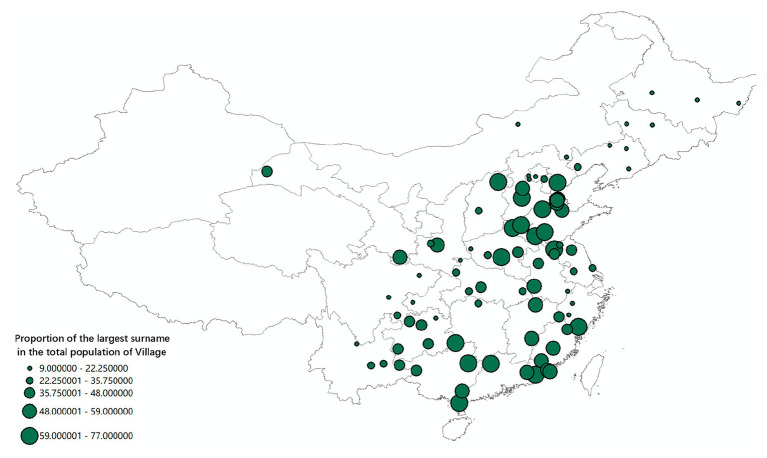
Regional Comparison of Lineage Development in China (2006).

The spatial variations of lineage culture and I/C are consistent. The spatial distribution of the divorce rate (see Figures 1, 2), independence of young offspring (see Figures 3, 4), fertility pattern (see Figures 5, 6), family structure and intergenerational relationship pattern (see Figures 7, 10), and lineage development (see Figures 8, 9) consistently support this conclusion. Northeast China and the Yangzi River Basin have been more susceptible to the individualist culture of the west since the 1980s because their cultural orientation was generally individualist long before that. In contrast, North China’s more developed lineage systems significantly weakened the influence of Western individualism. Finally, the historically strong lineage-based collectivism of South China was the most formidable obstacle to Western individualism.

The spatial correlation analysis of the spatial differences in lineage development and variables representing individualism establishes a firmer empirical basis for the above argument. We use all I/C indicators of each county located to the east of the Aihui-Tengchong Line as our dependent variables, and each county’s concentration of surnames (the population share of people with 20 most frequent surnames) as the explanatory variable. The spatial correlation analysis through the ArcGIS system demonstrates the strong explanatory power of surname concentration in accounting for the regional variation of I/C in China.

As the correlation coefficient matrix shows (see Table 3), the relationship between lineage and I/C variables is clear and significant. The lineage variable is positively correlated with family size and the proportion of three-generation families. As the level of lineage development of a region increases, family size and the proportion of extended families within all families also increases, and divorce rates fall. The lineage variable also has an interesting relationship with fertility patterns. The higher the lineage development, the higher the fertility rate, and the more likely people are to have more male children. The higher the lineage development level, the more likely the traditional family model is passed on from generation to generation.

**Table 3:** Correlation between various measured variables of I/C, lineage, rice, and climate demand-ıncome resource (CD-IR) factors in the year 2010.

	1	2	3	4	5	6	**7**	8	9	10	11	12
1. Family scale	1	−0.158^**^	0.608^**^	0.612^**^	0.072^**^	0.514^**^	0.145^**^	0.000	−0.082^**^	−0.153^**^	−0.109^**^	−0.163^**^
2. Percentage of individuals living alone	−0.158^**^	1	−0.338^**^	0.222^**^	0.280^**^	0.020	−0.106^**^	0.211^**^	0.127^**^	−0.106^**^	0.155^**^	0.036
3. Percentage of three-generation families	0.608^**^	−0.338^**^	1	0.341^**^	−0.177^**^	0.286^**^	0.144^**^	0.173^**^	−0.170^**^	−0.379^**^	−0.175^**^	−0.343^**^
4. Sex ratio at birth	0.612^**^	0.222^**^	0.341^**^	1	−0.057^**^	0.506^**^	0.327^**^	0.126^**^	0.022	−0.175^**^	−0.067^**^	−0.168^**^
5. Divorce rate	0.072^**^	0.280^**^	−0.177^**^	−0.057^**^	1	−0.129^**^	−0.205^**^	−0.100^**^	0.030	0.308^**^	0.209^**^	0.313^**^
6. Fertility rate	0.514^**^	0.020	0.286^**^	0.506^**^	−0.129^**^	1	0.146^**^	0.110^**^	−0.099^**^	−0.261^**^	−0.191^**^	−0.265^**^
7. Lineage development	0.145^**^	0.106^**^	0.144^**^	0.327^**^	−0.205^**^	0.146^**^	1	0.141^**^	0.078^**^	−0.087^**^	−0.039	−0.097^**^
8. Rice planting ratio	0.000	0.211^**^	0.173^**^	0.126^**^	−0.100^**^	0.110^**^	0.141^**^	1	0.061^**^	−0.570^**^	−0.016	−0.337^**^
9. Per capita GDP of 2010	−0.082^**^	0.127^**^	−0.170^**^	0.022	0.030	−0.099^**^	0.078^**^	0.061^**^	1	0.155^**^	0.151^**^	0.184^**^
10. Climatic Demands	−0.153^**^	−0.106^**^	−0.379^**^	−0.175^**^	0.308^**^	−0.261^**^	−0.087^**^	−0.570^**^	0.155^**^	1	0.266^**^	0.708^**^
11. Income Resources	−0.109^**^	0.155^**^	−0.175^**^	−0.067^**^	0.209^**^	−0.191^**^	−0.039	−0.016	0.151^**^	0.266^**^	1	0.785^**^
12. Income Resources * Climatic Demands	−0.163^**^	0.036	−0.343^**^	−0.168^**^	0.313^**^	−0.265^**^	−0.097^**^	−0.337^**^	0.184^**^	0.708^**^	0.785^**^	1

The correlation coefficient in the table is Pearson Correlation.

** Correlation is significant at the 0.01 level (two-tailed).

* Correlation is significant at the 0.05 level (two-tailed).

### Comparing the Explanatory Power of Different Theories

In order to compare the explanatory powers of the rice and lineage theories of I/C more systematically, we conducted additional quantitative analyses. After controlling for rice farming, CD/resource, and urbanization variables, we analyzed the lineage-related variables’ influence on I/C variables.

### Comparison Through a Regression Model

In the simple correlation matrix in Table 3, we find a significant correlation between the lineage factor and the variables that measure I/C. To further confirm the influence of lineage factors on I/C, we controlled factors that may significantly affect I/C. Existing studies have found that various factors, including pathogen, planting pattern, climate-demand, IR, and urbanization, cause regional differences in I/C. Models 4 and 5 are regression analysis models based on 2000 and 2010 county-level data on these critical factors. Although pathogen factors are essential, collecting county-level pathogen data in China is currently difficult. Therefore, the models mainly control rice, CD-IR, and urbanization factors.

Rice and CD-IR variables are highly collinear because climate affects both. Our collinearity test found a mean VIF = 3.65. At present, there are two main methods of resolving the collinearity problem. The first is to directly remove the less important factors in the regression equation. This approach, however, is not suitable for this study because we assess the validity of theories stressing the significance of rice and CD-IR factors. The second method uses variables that have collinearity. In time-series data, this can be done by subtracting the average value in a specific time range from observations. In space-series data, the average value within a specific spatial range is subtracted from observations (Rabe-Hesketh and Skrondal, 2008). We addressed the CD-IR factor by subtracting the average value of the provincial CD from the CD value of each county within the province, resulting in the differential value of the CD factor. We then incorporated this new value into the regression equation. We subtracted the provincial average from each county’s values because climate factors share strong similarities within a province. We could have also subtracted each prefecture-level city’s average value from each county’s CD value, but this would have been too cumbersome. Moreover, it may not significantly reduce collinearity because the range of prefecture-level cities is too small. After subtracting the provincial average values from counties’ CD values, the collinearity factor among the explanatory variables decreased significantly. The data analysis results (see Tables 4 and 5) show that, after controlling rice, CD-IR, and urbanization factors, the regression coefficients between lineage and I/C variables result in the following:

1. There is a negative correlation between lineage factors and the divorce rate (divorce rate representing individualism). Hence, the higher the region’s lineage development, the lower the divorce rate, and the lower the lineage development, the higher the divorce rate. We found this negative correlation for both 2000 and 2010, supporting our hypothesis (see Models 1 and 7).
2. Lineage development and the percentage of single-person households (such households representing individualism) tend to show a negative correlation in the 2000 data, which also supports our hypothesis (see Model 2). However, the correlation coefficient in the 2010 model does not support our hypothesis (see Model 8).
3. The degree of lineage development is positively correlated with family size (family size representing collectivism) for 2000 and 2010, which supports our hypothesis (see Models 3 and 9).
4. There is a positive correlation between the degree of lineage development and the proportion of three-generation direct families (such families representing collectivism). This correlation passed the significance test for the 2000 data (see Model 4). The correlation is also in line with the research hypothesis but did not pass the significance test for the 2010 data (see Model 10).
5. The positive correlation between the degree of lineage development and the fertility rate (see Models 5 and 11) and between lineage development and preference for male children (see Models 6 and 12) supports our hypothesis and also passes the significance test. As the degree of lineage development increases, people tend to have more children and are more inclined to have boys. This finding underlines the close relationship between familism and collectivism.
6. In the regression model, rice and CD-IR factors also have a certain degree of explanatory power. Rice theory argues that as the ratio of rice planting increases, the collectivist tendency increases. Models 1 and 7 show that the higher the proportion of paddy fields, the lower the divorce rate. In other words, the ratio of rice planting is negatively correlated with individualism. These findings are consistent with the rice theory. The rice factor also positively correlates with family size (see Models 3 and 5) and the ratio of three-generational families (see Models 4 and 8), variables representing collectivist tendencies. The rice theory can also explain fertility rate (see Models 5 and 11) and preference for male children (see Models 6 and 12). Hence, as the rice planting ratio increases, the fertility rate and preference for sons increase. However, like the lineage factor, the rice variable cannot predict the proportion of single-person households in a region. The regression coefficient does not conform to the rice theory’s assumptions. The CD-IR factor negatively correlates with the divorce rate (see Models 1 and 7), a factor representing individualism. It also exhibits a certain degree of positive correlation with other variables representing the collectivist tendency. However, after controlling for lineage and rice factors, most of the regression coefficients are relatively small.
7. Compared with other factors, such as divorce rate, fertility rate, and preference for sons (which all have a strong relationship with lineage factors for both 2000 and 2010), we find that variables measuring family structure and lineage factors do not have a stable relationship. For instance, Table 4 shows that the correlation coefficient between the proportion of three-generational households in 2000 (see Model 4) and the lineage factor is not significant. This may be explained by the fact that, with the help of census data, it is possible to accurately measure factors such as divorce rate, fertility rate, and preference for sons. However, it is not easy to measure family size and ratio of single-person and three-generational households in China, a country experiencing a rapid process of population migration and urbanization. Since family structure is continually changing, an accurate measurement of family size is difficult to obtain. For instance, measuring the ratio of three-generational families is challenging, because in many cases, the level of cohesion between parents and their married children may be high even when they do not live together. In other words, rapid rural outmigration makes measuring I/C based on family size and structure challenging. In contrast, measuring I/C based on the divorce rate, fertility rate, and preference for sons is easier and more reliable because migration does not affect these factors in the same way it affects family size and structure. In short, lineage, rice, and CD-IR factors can explain specific dimensions of I/C more accurately than others.

**Table 4:** The relationship between lineage development, rice production, and I/C indicators (2000).

	Model1	Model2	Model3	Model4	Model5	Model6
Variables	Divorce rate	Percentage of individuals living alone	Family scale	Percentage of three-generation families	Fertility rate	Sex ratio at birth
Lineage development	−0.194^***^	−0.0439^*^	0.0616^***^	0.0116	0.212^***^	0.378^***^
	(0.0270)	(0.0255)	(0.0214)	(0.0247)	(0.0235)	(0.0232)
Rice planting ratio	−0.0559^***^	0.196^***^	0.0567^***^	0.229^***^	0.0514^***^	0.196^***^
	(0.0215)	(0.0203)	(0.0170)	(0.0197)	(0.0187)	(0.0184)
Climatic Demands	−0.195^***^	−0.0309	0.0229	0.00276	−0.0548^**^	0.0706^***^
	(0.0256)	(0.0242)	(0.0203)	(0.0235)	(0.0223)	(0.0220)
Income Resources	−0.122^*^	0.0618	−0.257^***^	−0.154^**^	−0.417^***^	−0.0186
	(0.0664)	(0.0627)	(0.0527)	(0.0608)	(0.0577)	(0.0570)
Income Resources* Climatic Demands	0.0922	−0.00843	0.189^***^	0.0903	0.355^***^	−0.0145
	(0.0700)	(0.0661)	(0.0555)	(0.0642)	(0.0609)	(0.0601)
Proportion of non-agricultural population	0.133^***^	0.0269	−0.216^***^	−0.298^***^	−0.289^***^	−0.110^***^
	(0.0218)	(0.0206)	(0.0173)	(0.0200)	(0.0190)	(0.0187)
Observations	1,921	1,921	1,921	1,921	1,921	1,921
R^2^	0.084	0.053	0.105	0.175	0.181	0.203

Standard errors are in parentheses.

*** *p* < 0.01;

** *p* < 0.05;

* *p* < 0.1.

**Table 5:** The relationship between lineage development, rice production, and I/C indicators (2010).

	Model7	Model8	Model9	Model10	Model11	Model12
Variables	Divorce rate	Percentage of individuals living alone	Family scale	Percentage of three-generation families	Fertility rate	Sex ratio at birth
Lineage development	−0.153^***^	−0.0628^***^	0.104^***^	0.0714^***^	0.231^***^	0.255^***^
	(0.0517)	(0.0231)	(0.0229)	(0.0216)	(0.0222)	(0.0224)
Rice planting ratio	−0.0197	0.191^***^	0.0186	0.184^***^	0.0412^**^	0.107^***^
	(0.0462)	(0.0207)	(0.0204)	(0.0193)	(0.0198)	(0.0200)
Climatic Demands	−0.00981	0.0795^***^	0.0348	0.0338	−0.0461^*^	0.0383^*^
	(0.0551)	(0.0246)	(0.0244)	(0.0230)	(0.0236)	(0.0238)
Income Resources	−0.157	0.115^*^	−0.265^***^	−0.178^***^	−0.349^***^	−0.0693
	(0.142)	(0.0637)	(0.0629)	(0.0594)	(0.0611)	(0.0616)
Income Resources* Climatic Demands	0.147	−0.0776	0.197^***^	0.132^**^	0.294^***^	0.0167
	(0.150)	(0.0672)	(0.0664)	(0.0627)	(0.0645)	(0.0650)
Proportion of non-agricultural population	0.472^***^	0.368^***^	−0.248^***^	−0.352^***^	−0.274^***^	0.0249
	(0.0484)	(0.0217)	(0.0214)	(0.0202)	(0.0208)	(0.0210)
Observations	1,907	1,907	1,907	1,907	1,907	1,907
*R*^2^	0.057	0.167	0.099	0.211	0.170	0.085

Standard errors are in parentheses.

*** *p* < 0.01;

** *p* < 0.05;

* *p* < 0.1.

We conducted a factor analysis to comprehensively measure the explanatory power of these three factors. We generated collectivism and individualism factor scores through the principal component analysis of variables including family_scale, pro_of_three_gen, RSB, fertility, pro_of_single, and divorce. The data analysis results (see Table 6) show that the lineage factor has a statistically significant positive correlation with the collectivism factor (see Models 13 and 15) and a statistically significant negative correlation with the individualism factor (see Models 14 and 16). These findings support our hypothesis that the higher the lineage development, the higher the collectivist tendency and the lower the individualist tendency.

**Table 6:** Regression Analysis of Individualism and Collectivism Factor Scores and Each Variable.

	Model13	Model14	Model15	Model16
Variables	Collectivism of 2000	Individulism of 2000	Collectivism of 2010	Individulism of 2010
Lineage development	0.147^***^	−0.161^***^	0.201^***^	−0.0905^***^
	(0.0215)	(0.0260)	(0.0216)	(0.0218)
Rice planting ratio	0.0936^***^	−0.0948^***^	0.0845^***^	−0.0911^***^
	(0.0171)	(0.0207)	(0.0193)	(0.0195)
Climatic Demands	0.00341	−0.153^***^	0.0230	0.0392*
	(0.0204)	(0.0247)	(0.0230)	(0.0232)
Income Resources	−0.324^***^	−0.0410	−0.263^***^	−0.00578
	(0.0529)	(0.0639)	(0.0595)	(0.0600)
Income Resources* Climatic Demands	0.246^***^	0.0569	0.195^***^	0.0250
	(0.0558)	(0.0674)	(0.0629)	(0.0634)
Proportion of non-agricultural population	−0.317^***^	0.109^***^	−0.249^***^	0.484^***^
	(0.0174)	(0.0210)	(0.0203)	(0.0204)
Observations	1,921	1,921	1,907	1,907
*R*^2^	0.211	0.062	0.157	0.250

Standard errors are in parentheses.

*** *p* < 0.01;

** *p* < 0.05;

* *p* < 0.1.

Our lineage-based framework explains the differences in I/C scores for the year 2000 (see Models 13 and 14) and the regional differences in collectivism scores for the year 2010 (see Model 15). In these models, the lineage factor has relatively strong explanatory power, and the correlation coefficient is significantly greater than that of the rice factor. On the other hand, when explaining the individualism factor score for 2010, the correlation coefficient of the rice factor is slightly higher than that of the lineage factor (see Model 16). As shown in Table 6 models, the regression coefficients of the CD factor are the lowest. Hence, we found that the explanatory power of the CD factor is weaker than rice and lineage factors.

The correlation coefficients and directions of lineage and rice factors are highly consistent. Both factors explain I/C variation in China in a North–South axis. However, unlike the rice theory, our lineage-based approach separates the individualist Yangzi River Basin from collectivist South China. It also separates the individualist Northeast from the relatively collectivist North China. Therefore, compared with rice theory, the lineage theory provides a more detailed and optimized interpretation of regional variation of I/C in China.

The regional variation of I/C in China is not merely a North–South (or, more precisely, Qinling Mountains-Huai River) divide. There are significant cultural and psychological differences between North China, Northeast China, the Yangzi River Basin, and South China. This 4-fold regional variation has the potential to inform future studies of culture and psychology in China. It is in this sense that we believe that the rice theory is not wrong. Indeed, it should continue to inform us, though regional differences in Chinese culture and psychology should be further specified and related to the historical trajectories of migration and settlement of various Chinese regions.

## Conclusion

We began by pointing out the paradoxical conclusions of the rice theory and the climato-economic theory in their attempts to explain the regional variation of individualism–collectivism in China. Unlike existing studies, which use provinces as the unit of analysis, our study uses large-scale census data and social media data in order to analyze the regional distribution of individualism and collectivism in China from a more microscopic perspective at the county level. We find that the spatial distribution of I/C has apparent regional characteristics. South and North China are generally more inclined to collectivism, whereas the Yangzi River Basin and Northeast China are more inclined to individualism. These findings are significantly different from the claims of rice theory and climato-economic theory.

We offer the alternative explanation that different degrees of lineage development can account for the variation of individualism and collectivism in different Chinese regions. We show that regional cultural differences are related to regional differences in the degree of lineage development. Different regions in China have different lineage development trajectories due to their varied social and political histories, as well as migration and population settlement patterns. China’s historical background has resulted in significant regional variations of cultural orientations in recent decades, during which Western individualist values have affected China more than ever. While collectivist culture has resisted Western-style individualism in some regions, others have adopted it without much resistance. Collectivist culture has been strongest in South China and relatively strong in North China because of earlier population settlement, which allowed full development of lineage organizations. In contrast, the Yangzi River Basin and Northeast China have maintained a fairly strong individualist culture due to the relatively late population settlement and weak lineage organizations. This lineage-based framework makes a novel theoretical contribution to cross-cultural psychology, particularly in studies of Chinese culture.

We want to point to some limitations of this research. Talhelm et al. (2014) measure the rice farming ratio at the provincial level. We carry out a county-level analysis. There are more than 2,000 counties in China, and it is almost impossible to find data on the ratio of paddy fields to total cultivated land for all counties. Therefore, we used the indicator of the ratio of rice sown area to grain sown area provided by the first agricultural census (1996) to calculate the rice farming ratio for each region. This procedure is slightly different from the measurement method in the paper by Talhelm et al. (2014). This difference may have a specific impact on the research conclusions. However, we think that the level of capability of these two measurement methods is not very different.

There are certain disagreements between the rice theory and the climato-economic theory. Although our conclusions are different from both approaches, overall, we agree more with the rice theory. For example, similar to Talhelm et al. (2014), we also found that rice farming region to the south of the Qinling Mountains and Huai River is an area with a relatively high level of lineage development. Moreover, both our lineage-based approach and rice theory describe the Yangzi River Basin as a cultural region that is different from both North China and South China.

Like the rice theory, our analysis is significantly different from the findings of the climato-economic theorists. Our differences may be due to our different units of analysis. While the climato-economic scholarship examines the regional variation of I/C in China at the provincial level, we carried out a county-level analysis, which is more granular. However, this is not the end of this debate. More data sources will become available in the future, especially with the further development of the collection and analysis of big data. Hence, we need to continue testing our theories by using these data sources.

Further research is required to explore various dimensions of this issue. Multiple factors affect the cultural-psychological orientations of modern Chinese society, the lineage development in the ancient period being just one of them. Existing studies have revealed that differences in ecosystems, wealth levels, and other factors are critical in explaining contemporary cultural orientations. This study does not analyze these variables due to space limitations. I/C is only one among the five dimensions of cultural variation formulated by Hofstede (2011). The other four dimensions are power distance, masculinity/femininity, uncertainty avoidance, and long-term vs. short-term orientation. Similarly, in addition to the I/C dimension, the three-dimensional perspective Triandis et al. (1988) includes tightness/looseness and cultural complexity (Chua et al., 2019; Talhelm and English, 2020). The effects of lineage development should be explored from all these dimensions. The relationship between lineage development and different styles of thinking (analytic/holistic) also deserves further research.

Finally, the effects of migration on cultural change should also be taken seriously. The ratio of the urban population in China rose from 35.8% in 2000 to 60.3% in 2019 (World Bank, n.d.). Moreover, there are 291 million migrant workers in China who left their villages to work in urban areas within their home province or other provinces (National Bureau of Statistics of China, 2020). Recent studies of domestic educational migration in China show that cultural differences along the North–South axis significantly shape the coping strategies of young people who migrate from/to the North/South for university education. These studies stress that coping strategies based on change of environment (primary coping) are prevalent in North China, and those based on fitting into the environment (secondary coping) are prevalent in South China. Chinese youth who do not cross the North–South border do not experience significant acculturative stress (English et al., 2019). However, those crossing the North/South border for university education do. Coping strategies learned from home are highly important when coping with this stress (English and Worlton, 2017; English and Geeraert, 2020). Hence, future studies of regional variation of culture in China should seriously consider the effects of migration.

## Author Contributions

All authors contributed to the article and approved the submitted version.

### Conflict of Interest

The authors declare that the research was conducted in the absence of any commercial or financial relationships that could be construed as a potential conflict of interest.

## Footnotes

^1^To control the floating population’s influence, we use the registered population as the standard in the calculation process. China’s census simultaneously counts both the current resident population and the registered population. The statistics on current residence include the migrant population, while household registration statistics mainly indicate a person’s birthplace or area, where a person has lived since childhood. Since most migrant workers are still not entitled to a pension that would allow them to stay in the cities after retirement, those working in cities generally do not change their household registration and usually eventually return to their rural residences.

^2^We have included parts of Yunnan and Guizhou into the Yangtze River Basin. Although Yunnan and Guizhou are in Southwestern China, their cultural characteristics have notable similarities with the Yangzi River Basin. However, the cultural formation of these regions may be different from the pattern of the Yangzi River Basin. In our future research, we plan to investigate the reasons of the similarities of these two areas and explore whether or not Southwestern China constitutes a distinct cultural region.

^3^Other sample survey data, such as neighborhood-level data of the China General Social Survey (CGSS2010) and China Family Panel Studies (CFPS2010), also measure the concentration of surnames at the village level. The data analysis yield roughly similar results to our analysis of the CGSS2006 data.
