# Supplementary material for: The Lineage Theory of the Regional Variation of Individualism/Collectivism in China
Source: Front Psychol. 2021 Jan 20;11:596762. doi: 10.3389/fpsyg.2020.596762 (PMC7854463; doi:10.3389/fpsyg.2020.596762)
Supplement: Supplementary file 4 [file Table_4.docx]

Supplementary Materials

**The interpretation of lineage variables based on experimental psychological data**

In the main text, we use regression equations to analyze lineage variables to explain the regional variation of divorce rate, family structure, offspring independence, fertility rate, and other indicators. We analyze these indicators at the county level. After that, we use the original data of Talhelm et al.'s study (2014) to assess the explanatory power of lineage variables in the analysis of the individuals' psychological-behavioral tendencies.

**Table 1. The degree of lineage development, rice farming, and the width of self**

|  | **Beta** | **t** | **Sig.** | **Beta** | **t** | **Sig** |
| --- | --- | --- | --- | --- | --- | --- |
| Gender2 | -.180 | -4.787 | .000 | -.100 | -2.367 | .018 |
| xeperzhang | -.502 | -12.346 | .000 | -.540 | -12.034 | .000 |
| Lineage development | -.159 | -3.915 | .000 | -.107 | -2.215 | .027 |
| Rice planting ratio |  |  |  | -.158 | -3.394 | .001 |
| Climatic Demands |  |  |  | .046 | .869 | .385 |
| Income Resources |  |  |  | .108 | .766 | .444 |
| Income Resources * Climatic Demands |  |  |  | -.078 | -.547 | .584 |
|  | R^2^=0.258 | | | R^2^=0.269 | | |

‘xeperzhang’ is a dummy variable about location in the original supplymentary data provided by Talhelm et al.'s study (2014).

Dummy variable for location

**Table 2. The degree of lineage development, rice farming, and** **the width of friends**

|  | **Beta** | **t** | **Sig.** | **Beta** | **t** | **Sig** |
| --- | --- | --- | --- | --- | --- | --- |
| Gender2 | -.116 | -2.890 | .004 | -.100 | -2.367 | .018 |
| xeperzhang | -.427 | -9.834 | .000 | -.448 | -9.332 | .000 |
| Lineage development | -.165 | -3.812 | .000 | -.127 | -2.462 | .014 |
| Rice planting ratio |  |  |  | -.195 | -3.917 | .000 |
| Climatic Demands |  |  |  | -.048 | -.853 | .394 |
| Income Resources |  |  |  | .171 | 1.132 | .258 |
| Income Resources * Climatic Demands |  |  |  | -.108 | -.715 | .475 |
|  | R^2^=0.175 | | | R^2^=0.184 | | |

The primary conclusion of our analysis of Talhelm et al.'s raw data is that the width of self and friends' width is significantly narrower in people living in regions with strong lineages than those living in regions where lineages are weak. Hence, people in lineage areas tend to view themselves or other individuals as relatively unimportant. The individual is only a part of the lineage group and does not have significant importance outside the lineage.

On the contrary, in areas where lineage development is weak, because there is no such external structural constraint as lineage, individuals in the community have migrated from all directions. The community does not have much blood ties and cultural obligations. In other words, the community has atomistic characteristics.

Compared with individuals within lineage groups, individuals who grew up in atomistic communities are relatively "unfettered," "freely stretching" .As a result, the degree of individual expansion far exceeds that of lineage areas.

Similarly, we found that individuals in lineage areas tend to view their friends as less important than individuals living in non-lineage regions. This finding is probably related to the fact that individuals who have grown up in a lineage-dominated environment have a relatively small view of themselves and others.

Figure 1 illustrates these findings.


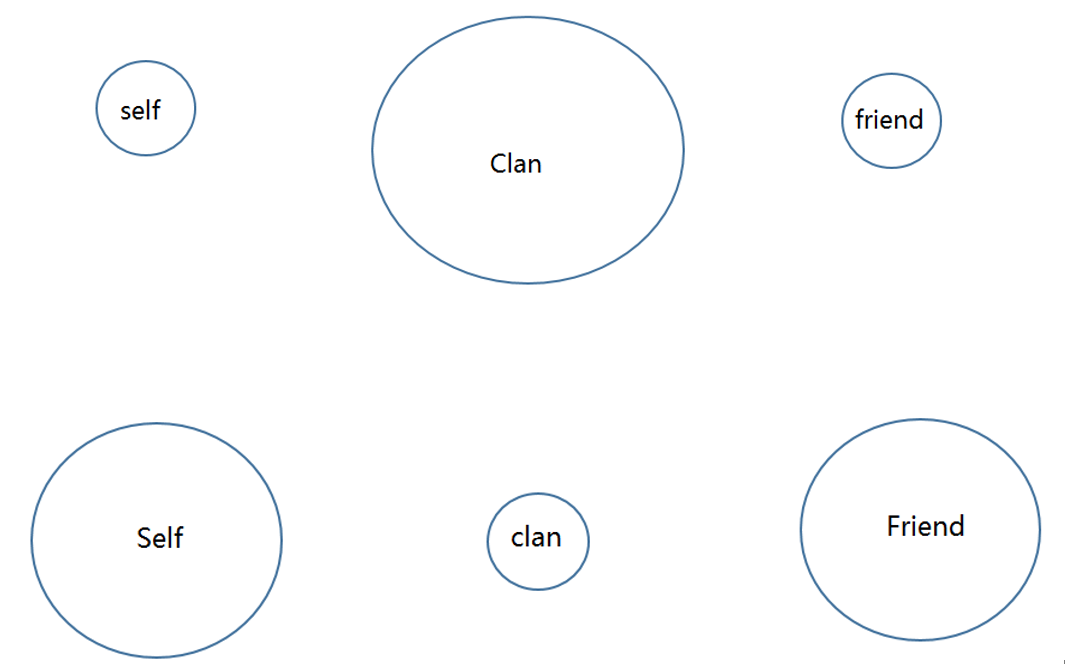


Figure 1. Clan, self, and friends
